# The Trajectory of Gene Discovery in Retinitis Pigmentosa

**DOI:** 10.3390/genes17080940

**Published:** 2026-08-12

**Authors:** Anthony X. J. Wong, Zachary Chua, Jing Guo, Hwee Goon Tay, Zhen Xun Wang, Mathieu Quinodoz, Tien-En Tan, Carlo Rivolta, Beau J. Fenner

**Affiliations:** 1 Ocular Genetics Service, Singapore National Eye Centre, 11 Third Hospital Avenue, Singapore 168751, Singapore; 2 Singapore Eye Research Institute, Singapore 168751, Singapore; 3 Ophthalmic Genetics Group, Institute of Molecular and Clinical Ophthalmology Basel (IOB), Mittlere Str. 91, 4031 Basel, Switzerland; 4 Department of Ophthalmology, University of Basel, Mittlere Str. 91, 4056 Basel, Switzerland; 5 Department of Genetics and Genome Biology, University of Leicester, Leicester LE1 7RH, UK; 6 Department of Surgical Retina, Singapore National Eye Centre, 11 Third Hospital Avenue, Singapore 168751, Singapore; 7 Department of Medical Retina, Singapore National Eye Centre, 11 Third Hospital Avenue, Singapore 168751, Singapore

**Keywords:** retinitis pigmentosa, inherited retinal disease, gene discovery, retinal dystrophy, genetic diagnosis, genotype–phenotype correlation, age of symptom onset, unsolved inherited retinal disease

## Abstract

**Background:** To assess how historical patterns in retinitis pigmentosa (RP) gene inheritance, functional category, and phenotypic onset may inform the remaining unsolved RP discovery space. **Methods:** We manually reviewed PubMed-indexed primary reports in the RetiGene database, supplemented with PubMed-indexed human studies where necessary to improve extractable phenotype data. Gene-level variables extracted included age of symptom onset, inheritance pattern, functional category and syndromic association. Gene-level associations between earliest year of RP gene discovery and mean age of symptom onset were assessed, alongside temporal analyses for functional category, inheritance, and syndromic patterns. **Results:** Later year of gene discovery was associated with later mean age of symptom onset, both in the primary analysis restricted to genes with at least 5 extractable onset cases (*n* = 83, *p* < 0.001) and in a stricter sensitivity analysis restricted to genes with at least 10 onset cases (*n* = 66, *p* = 0.00525). Year of discovery differed significantly across functional categories (*p* < 0.001). No significant temporal association was observed for AR versus non-AR inheritance or syndromic status. **Conclusions:** This hypothesis-generating analysis suggests that observed patterns likely reflect a combination of biological detectability, study design, and publication dynamics rather than intrinsic gene–phenotype relationships alone; within this context, future RP-solving efforts may benefit from greater attention to less obvious biological pathways (such as ciliary, transport, and metabolic categories), along with later-presenting or less conspicuous phenotypes.

## 1. Introduction

Despite major advances in next-generation sequencing, a substantial proportion of inherited retinal disease (IRD) cases remain genetically unsolved. A recent systematic review and meta-analysis estimated current diagnostic yields for IRD at approximately 40–75%, with pooled yield for more recent mixed-phenotype cohorts around 64.2%, indicating that a large unresolved burden persists even in the genomic era [1,2,3,4,5]. Importantly, solve rates vary markedly across cohorts and disease contexts. In Japan, large studies have reported diagnostic rates of roughly 40% [6,7], while some Western cohorts have reported rates above 60%, including approximately 70% in the Irish Target 5000 programme, 67.4% in Scotland, and 67.6% after stepwise personalised genomic re-evaluation in one recent series [3,8,9]. Earlier reviews have also argued that many of the missing diagnoses are likely to reside in known IRD genes but remain undetected or misinterpreted because of technical and analytic limitations [10,11,12]. These differences likely reflect not only geography, but also phenotype composition, family structure, testing strategy, and variant interpretation frameworks.

Retinitis pigmentosa (RP) remains particularly relevant because it is the most common and genetically heterogeneous of all IRD phenotypes, and recent work suggests that more narrowly defined or clinically distinctive IRD phenotypes may achieve higher diagnostic yield than broader categories such as RP [1,13,14]. As the rate of discovery of IRD genes has slowed in recent years [10,11], it becomes increasingly important to understand what features may characterise the remaining unsolved space to help future solving efforts and facilitate improved patient counselling and therapeutic planning [15]. This raises not only the question of how many RP genes have been identified, but also what kinds of genes were discovered earlier, what kinds emerged later, and what this trajectory may imply about the genes and mechanisms that remain unresolved. Earlier phases of RP gene discovery were enriched for genes associated with severe, recognisable, or mechanistically intuitive disease, often driven by linkage and positional cloning in large pedigrees and clinically distinctive disorders, while later phases have depended on broader sequencing strategies such as exome or genome sequencing, and improved interpretation of non-coding, structural, and splice-altering variants [5,16,17]. The discovery timeline itself may therefore offer indirect clues to the phenotypes, pathways, and genomic architectures that continue to occupy the unsolved space. This question is particularly relevant in a field where curated gene resources such as RetiGene have emerged in response to the increasing scale, complexity, and continued incompleteness of RP genetics [18].

Hence, we sought to examine how inheritance pattern, protein function, and phenotypic onset timing and syndromic association varied across the RP discovery timeline, and how this might help inform where future gene discovery efforts are most likely to yield additional insight in unsolved cases of RP. However, gene discovery chronology reflects not only underlying disease biology but also the evolution of genomic technologies, study design, and research priorities. Temporal patterns in gene discovery should therefore not be interpreted as purely biological signals, but as a composite of biological detectability, technological capability, and ascertainment context. The present study is thus intended as a hypothesis-generating analysis of discovery patterns rather than a definitive causal model of disease architecture.

## 2. Methods

### 2.1. Study Design and Data Collection

This study was a retrospective, literature-derived, gene-level observational analysis of RP gene discovery trends, based on extraction of predefined gene-level variables from eligible RetiGene-linked primary reports [18]. Parameters already included in the RetiGene database included year of earliest primary report associated with RP gene taken as discovery year, broad inheritance patterns and syndromic associations for each gene. Inheritance categories reflected the current curated modes of inheritance associated with each gene rather than only the mode reported at the time of its initial RP association.

The RetiGene-linked PMID indices were reviewed manually for phenotypic data extraction and deduplicated. As RetiGene-linked primary reports did not always contain sufficient extractable human RP-specific age-of-onset data, supplementary targeted PubMed searches were performed on a gene-by-gene basis using combinations of gene name with “retina,” “cohort patients,” and “retinitis pigmentosa.” Searches were reviewed chronologically to identify additional eligible human primary reports with extractable age-of-symptom-onset data. These searches were intended to augment phenotype extraction rather than to constitute a separate exhaustive systematic search stream, and to improve the stability of gene-level age-of-onset estimates while retaining adequate representation across the RP discovery timeline, with ≥5-case and ≥10-case thresholds used for the primary and sensitivity analyses, respectively. Cases were included when the source article provided sufficient genotypic confirmation and extractable phenotype data for analysis. Articles were excluded if they were non-primary reports, lacked extractable genotypic or phenotypic data, did not provide RP-specific clinical information, or represented duplicate or overlapping case reports. Cases were cross-checked using available identifiers including gene, variant, pedigree structure, ethnicity, reporting institution, and clinical descriptors.

Because gene inclusion was based on the RetiGene database, which reflects expert curation and evolving definitions of RP-associated genes, the dataset may incorporate selection biases related to gene classification and inclusion criteria rather than representing an entirely neutral or exhaustive gene set.

RetiGene was selected as our primary framework for gene inclusion and analysis as it provides an expert-curated atlas of IRD-associated genes with literature-linked evidence and temporal information on gene discovery. This structure was particularly suited to the present study, which required identification of RP-associated genes together with their earliest reported RP association. RetNet and Online Mendelian Inheritance in Man (OMIM) were used as complementary reference resources to corroborate gene function and retinal disease associations [19,20]. The NIH eyeGENE resource was not used because it was developed principally as a patient-level genotype-phenotype database, registry and DNA repository for inherited eye diseases, as opposed to a comprehensive historical catalogue of RP gene discovery [21].

### 2.2. Variables Extracted and Definitions

Extracted variables included gene identity, year of discovery, inheritance pattern, functional category, syndromic association and age of symptom onset. Year of gene discovery was operationally defined as the publication year of the earliest primary report establishing an association between pathogenic variation in that gene and an RP phenotype, as identified through the RetiGene-linked literature. This did not refer to the year in which the gene itself was first discovered, nor to its first association with any retinal or systemic disease.

Age of symptom onset was defined as the earliest reported age at which visual symptoms or dysfunction were described, where available. To enable inclusion of descriptively reported onset data in quantitative analysis, “childhood onset” was coded as 4 years, and “infancy onset” as 0.5 years, allowing non-exact onset categories to be represented numerically while acknowledging their inherent imprecision. These transformations were applied to enable approximate ranking of onset timing across genes rather than precise quantitative estimation. Given heterogeneity in reporting (e.g., symptom onset vs. diagnosis, nyctalopia vs. visual acuity decline), these values should be interpreted as coarse proxies of relative onset timing rather than exact measures. Average values were used where age ranges were provided. Functional categories of each gene were corroborated with established inherited retinal disease reference resources RetNet and OMIM [19,20], reflecting the principal known biological role of each gene in retinal physiology or pathogenesis. Where genes had multiple reported functions, the category most consistently linked to retinal pathophysiology in reference sources was assigned. Variables not consistently reported in the source literature were treated as missing.

Syndromic-association status was derived from the broad phenotype category in the original dataset. Genes that were classified as “Both” reported associations with both syndromic and non-syndromic retinal phenotypes, hence were considered genes with syndromic associations rather than exclusively syndromic genes. Genes classified as “Non-syndromic” had no recorded syndromic association in the dataset.

### 2.3. Statistical Analysis

Analyses were conducted at the gene level, as year of discovery is a gene-level attribute and published case counts vary substantially across reports because of publication practices, pedigree structure, and evidence standards. Descriptive statistics were generated for the literature-derived dataset, including the number of genes identified across discovery eras, inheritance categories, functional categories, and phenotypic onset. Continuous variables were summarised using mean, standard deviation, median, and range as appropriate, while categorical variables were reported as counts and percentages.

The primary temporal analysis examined associations between discovery timing and gene-level inheritance, functional category, and age of symptom onset. Gene-level associations between year of gene discovery and age of symptom onset were assessed using Pearson correlation and linear regression. For these analyses, mean age of symptom onset was calculated for genes with at least 5 cases of extractable onset data. To assess the robustness of the observed association, a sensitivity analysis was performed by repeating the Pearson correlation and linear regression analyses after restricting the dataset to genes supported by at least 10 cases with extractable age-of-symptom-onset data. These support thresholds were selected to reduce the influence of sparsely supported gene-level estimates while retaining adequate representation across the RP discovery timeline.

Differences in year of discovery across functional categories were assessed using one-way analysis of variance and Kruskal–Wallis testing. Inheritance pattern was summarised descriptively across all 105 genes, including mixed-inheritance categories. For inferential analysis of temporal inheritance trends, mixed-inheritance genes were excluded, and the remaining genes were dichotomised as autosomal recessive versus non-autosomal recessive, leaving 96 genes for analysis. Temporal differences in the distribution of autosomal recessive versus non-autosomal recessive genes across discovery eras were assessed using chi-square testing.

As a supplementary gene-level analysis, genes were classified as having syndromic associations if they were marked as having a systemic or syndromic association in the original dataset. The association between syndromic-association status and year of discovery was assessed using point-biserial correlation, while differences in the proportion of genes with syndromic associations across discovery eras were assessed using chi-square testing. This analysis was intended to assess whether genes with syndromic associations showed any temporal pattern across the RP discovery timeline, and to identify the discovery eras in which they were most concentrated. Furthermore, to assess whether the observed association between discovery year and mean age of symptom onset was influenced by genes with syndromic associations, the primary ≥5-case analysis and ≥10-case sensitivity analysis were additionally repeated after stratification by syndromic-association status. A linear regression model that incorporated discovery year, syndromic-association status and a discovery year x syndromic-association status interaction term was used to assess if temporal associations differed significantly between groups.

All statistical analyses were performed using IBM SPSS Statistics version 31.0.2.0 and Python 3.13.5. A two-sided *p*-value of less than 0.05 was considered statistically significant.

Because analyses were conducted at the gene level, all inferences represent gene-level (ecological) associations rather than patient-level effects. This approach treats each gene as a single unit regardless of the number of reported cases, which may introduce aggregation bias and obscure within-gene phenotypic variability. Consequently, findings should be interpreted as reflecting broad trends in gene discovery rather than precise estimates of phenotype–genotype relationships at the individual level.

## 3. Results

**Figure 1:**
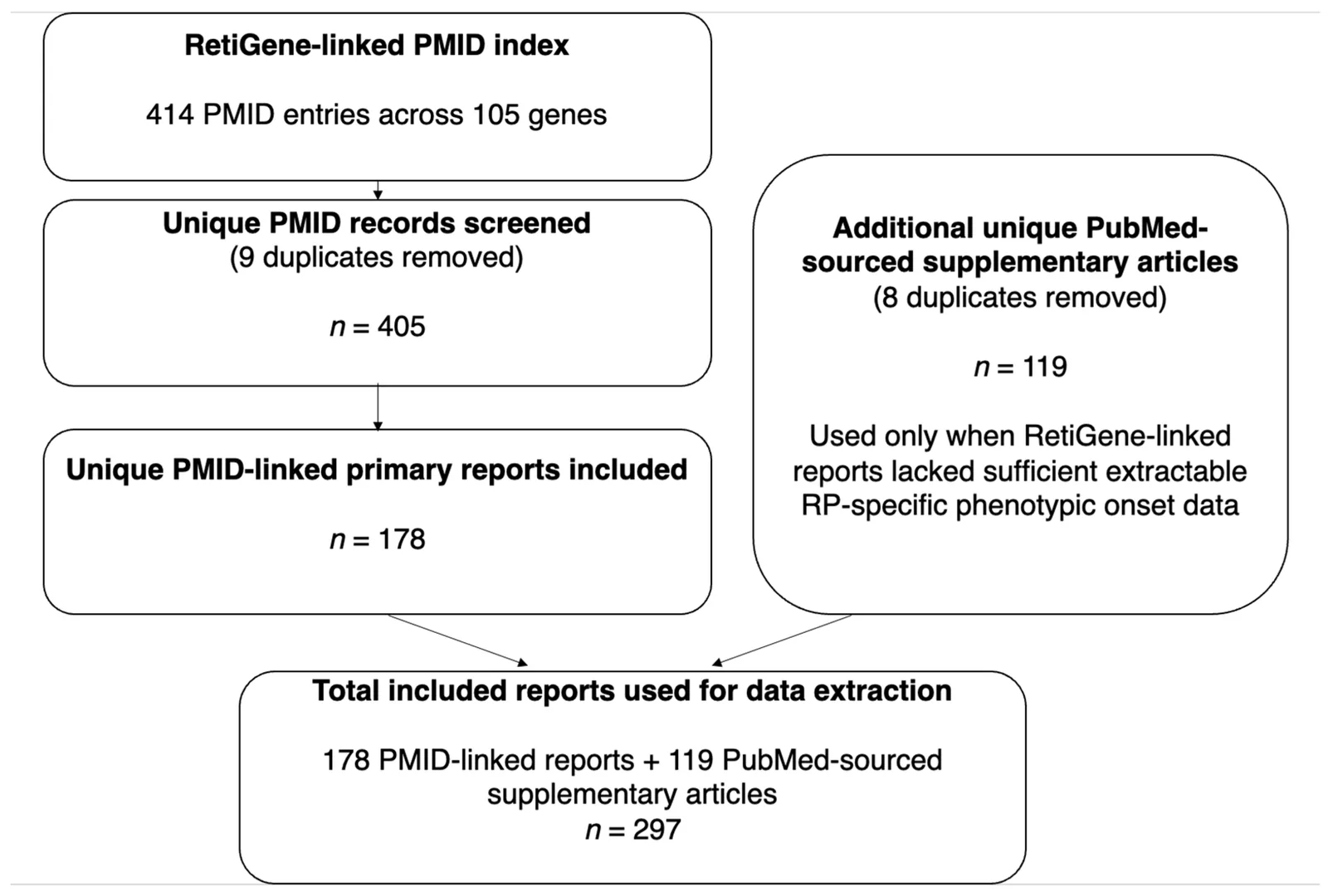
Study selection flowchart. The RetiGene-linked PMID index comprised 414 entries across 105 RP-associated genes. After removal of 9 duplicate entries, 405 unique PMID records remained for screening, of which 178 PMID-linked primary reports met eligibility criteria for phenotypic data extraction. Supplementary PubMed searches contributed 127 unique PMIDs, of which 119 were additional unique reports beyond the RetiGene-linked phenotype-extraction set. Overall, 297 unique PMID-indexed reports contributed to phenotype extraction. Discovery year was assigned from the earliest RP-associated report for each gene, with a total of 102 out of 105 genes with extractable age-of-symptom-onset data.

The RetiGene-linked PubMed index comprised 414 PMID entries across 105 genes. After deduplication, 405 unique PMID records remained for screening, of which 178 PMID-linked primary reports met eligibility criteria for phenotypic data extraction. As some RetiGene-linked reports did not provide sufficient extractable human RP-specific clinical or variant data, 119 unique supplementary articles were included for additional gene-level phenotype characterisation. Overall, 297 unique primary reports representing 102 RP-associated genes published between 1990 and 2025 were included in the final analysis for age-of-symptom onset extraction (Figure 1). The remaining genes lacked sufficient extractable age-of-onset data and were included in descriptive but not onset analyses.

**Figure 2:**
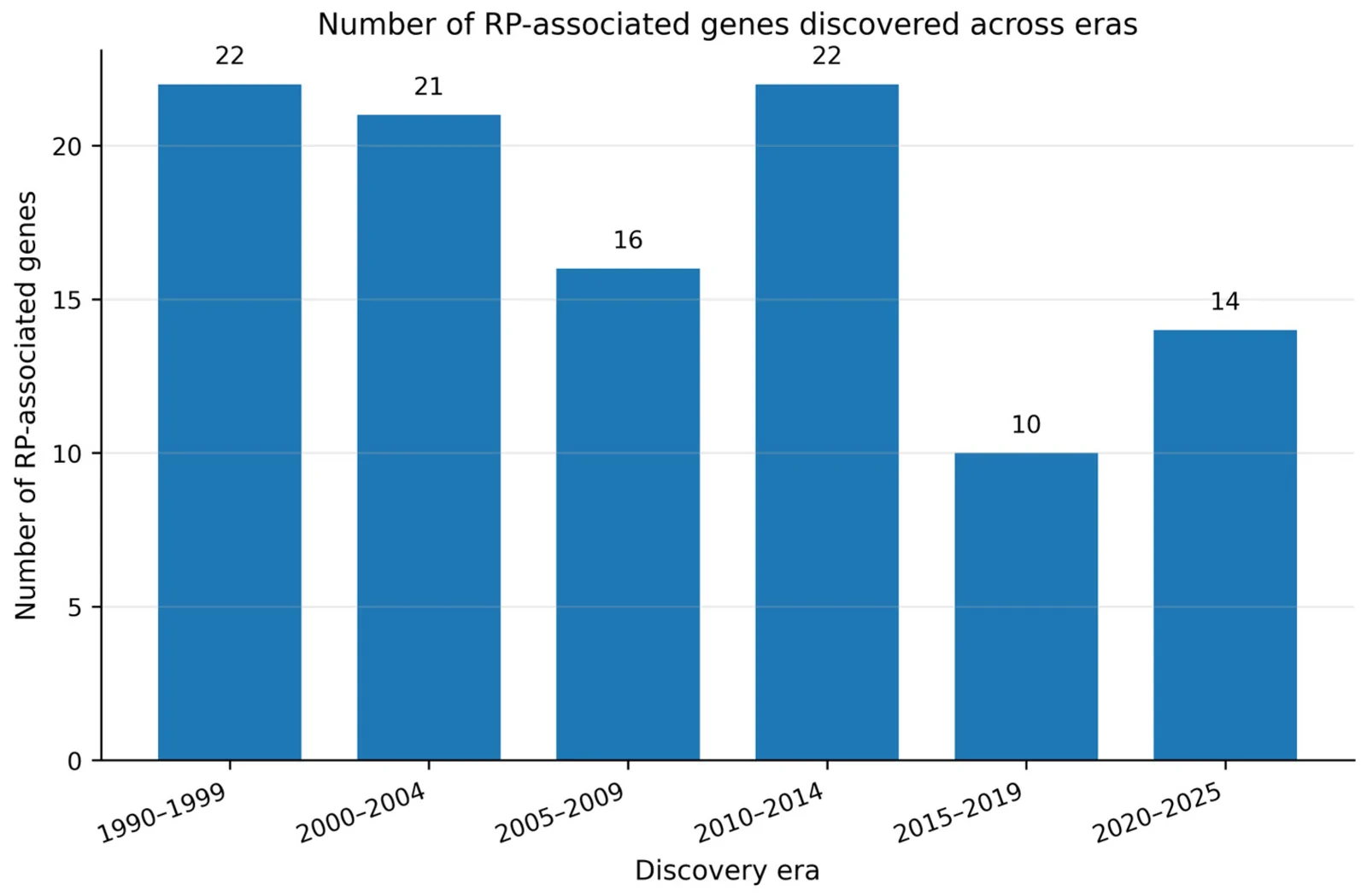
Number of RP-associated genes discovered across eras. Bar chart showing the number of RP-associated genes identified in each discovery era from 1990 to 2025. Gene discovery was distributed across all eras, with peaks in 1990 to 1999 and 2010 to 2014.

A total of 105 RP-associated genes were identified across the discovery timeline from 1990 to 2025, with the largest number of genes discovered in 1990 to 1999 and 2010 to 2014 (22/105 across each discovery period) (Figure 2).

Descriptively, autosomal recessive genes dominated across all RP gene discovery eras (77/105 genes; 73.3%), while autosomal dominant (14/105 genes; 13.3%), X-linked (5/105 genes; 4.8%), and mixed inheritance (9/105 genes; 8.6%) categories were less frequent (Table 1). The proportion of autosomal recessive genes appeared highest in the 2010 to 2019 discovery periods (90.9% in 2010–2014 and 90.0% in 2015–2019), although this pattern was less marked in the most recent era (71.4% of all genes discovered).

To assess if there was a shift toward autosomal recessive inheritance with increasing year of gene discovery, mixed-inheritance genes were excluded for inferential analysis, leaving 96 genes for AR versus non-AR comparison (Table 2). AR genes comprised 70.6% of genes in 1990–1999 (12/17), 77.8% in 2000–2004 (14/18), and 80.0% in 2005–2009 (12/15). This enrichment persisted in later periods, with AR genes accounting for 90.9% in 2010–2014 (20/22) and 90.0% in 2015–2019 (9/10), before returning to 71.4% in 2020–2025 (10/14). Overall, AR genes represented 80.2% of genes across eras (77/96), compared with 19.8% non-AR (AD/XL) genes (19/96). Although autosomal recessive genes predominated across all discovery eras, the proportion of AR versus non-AR genes did not differ significantly across eras (Pearson chi-square *p* = 0.560).

**Figure 3:**
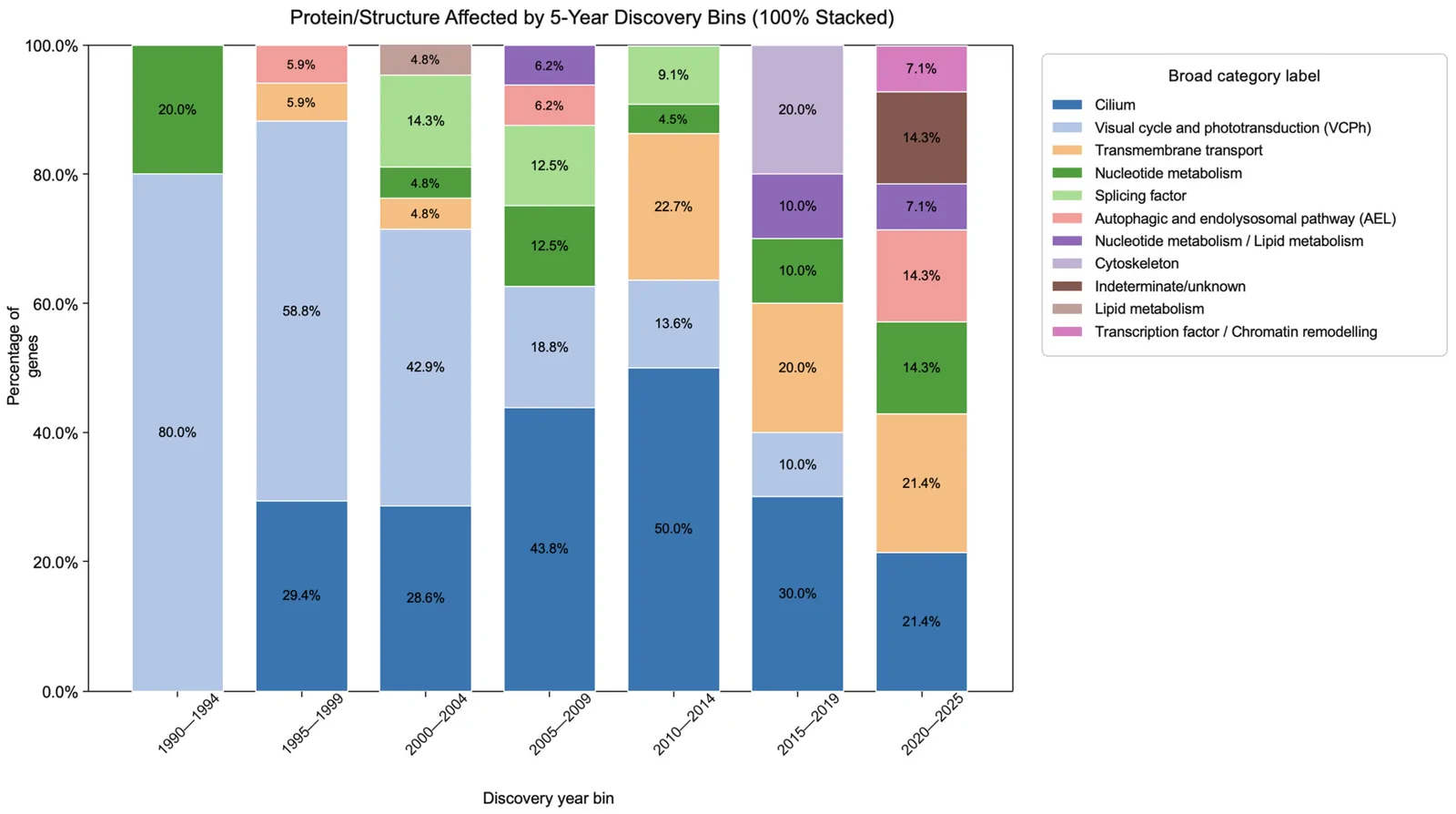
**Functional categories across discovery eras.** Stacked proportional bar chart showing the distribution of RP-associated genes by broad functional protein category within each discovery era. Earlier periods were relatively enriched for visual cycle and phototransduction genes, whereas later periods showed broader representation of ciliary, transport, metabolic, and less-defined categories. Gene counts for each era are shown above the bars.

The functional composition of RP gene discovery changed across eras, with earlier periods relatively enriched for visual cycle and phototransduction genes (e.g., *RHO*, *PDE6A*, *PDE6B*, *CNGA1*, and *SAG*) and later periods showing broader representation of ciliary, transport, metabolic, and less-defined categories (e.g., *BBS1*, *BBS2*, *CFAP418*, *COQ8B* and *IDH3B*) (Figure 3). The full list of genes and their corresponding functional categories is provided in Table 3.

Year of discovery differed across functional categories (ANOVA *p* = 4.26 × 10^−6^; Kruskal–Wallis *p* = 5.98 × 10^−5^) (Table 3). Genes involved in visual cycle or phototransduction were identified earliest overall, with a mean discovery year of 2001 (median 2000; range 1990–2016). Splicing-factor genes and ciliary genes tended to be discovered later, with mean discovery years of 2007 and 2009, respectively. Categories identified later still included nucleotide metabolism genes (mean 2011), autophagolysosomal genes (mean 2012), and transmembrane transport genes (mean 2013). The most recently discovered categories were nucleotide/lipid metabolism (mean 2016), cytoskeletal genes (mean 2016), transcription factor genes (mean 2022), and unmapped or unknown genes (mean 2023), although some of these categories contained very small numbers of genes.

Early discovery bins were dominated by canonical visual cycle/phototransduction (VCPh) genes, comprising 80.0% of genes in 1990–1994 (4/5) and 58.8% in 1995–1999 (10/17), before declining progressively across subsequent eras (42.9% in 2000–2004; 18.8% in 2005–2009; 13.6% in 2010–2014; 10.0% in 2015–2019) and reaching 0% in 2020–2025 (0/14) (Table 4). In contrast, ciliary pathway genes increased in relative representation after the earliest era, rising from 0% in 1990–1994 (0/5) to 29.4% in 1995–1999 (5/17) and 28.6% in 2000–2004 (6/21), peaking in 2010–2014 at 50.0% (11/22), and remaining prominent thereafter (30.0% in 2015–2019; 21.4% in 2020–2025). Later eras also showed greater representation of non-VCPh categories, including transmembrane transport genes (22.7% in 2010–2014; 20.0% in 2015–2019; 21.4% in 2020–2025) and nucleotide metabolism genes (12.5% in 2005–2009; 10.0% in 2015–2019; 14.3% in 2020–2025).

**Figure 4:**
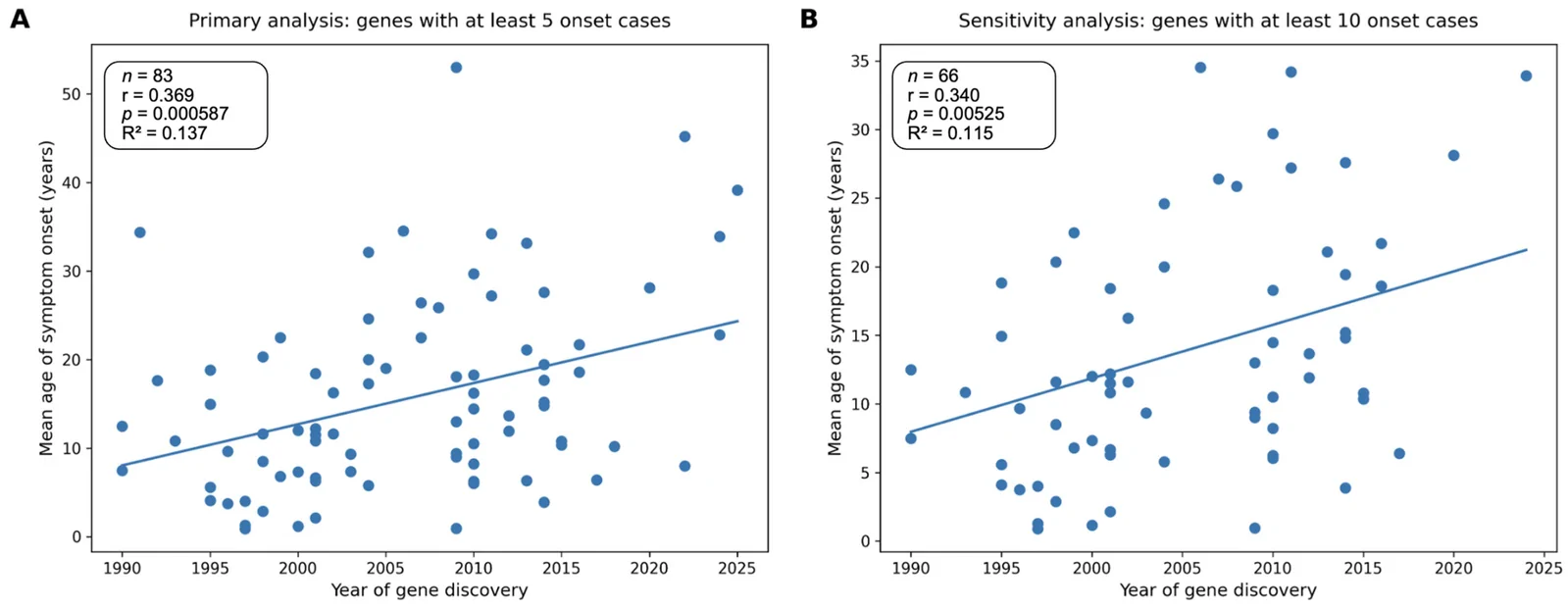
**Gene-level association between year of gene discovery and mean age of symptom onset at different support thresholds.** (**A**) Primary analysis restricted to genes with at least 5 extractable onset cases per gene (*n* = 83, r = 0.369, *p* = 0.000587, R^2^ = 0.137). (**B**) Sensitivity analysis restricted to genes with at least 10 extractable onset cases per gene (*n* = 66, r = 0.340, *p* = 0.00525, R^2^ = 0.115).

Age-of-symptom-onset analyses were performed at the gene level using mean onset age per gene. In the primary analysis comprising 83 genes with at least 5 extractable onset observations, later year of discovery was significantly associated with later mean age of symptom onset (Pearson r = 0.369, *p* = 0.000587, R^2^ = 0.137) (Figure 4). This association remained significant in a stricter sensitivity analysis restricted to genes with at least 10 extractable onset observations per gene (*n* = 66 genes, Pearson r = 0.340, *p* = 0.00525, R^2^ = 0.115), supporting the robustness of the overall temporal relationship, though the effect size was modestly attenuated relative to the ≥5-case subset. A gene-level support table summarising the number of cases contributing age-of-symptom-onset data for each gene is provided in Supplementary Table S1.

To further assess the potential influence of syndromic association on observed relationships between discovery year and age of symptom onset, the analysis was repeated after stratification by syndromic-association status. Among non-syndromic genes, later discovery year was significantly associated with later mean age of symptom onset both at the≥5-case threshold (*n* = 55, r = 0.470, *p* = 0.000295) and at the ≥10-case threshold (*n* = 47, r = 0.416, *p* = 0.00362). Conversely, no significant association was detected in genes with syndromic associations at either the≥5-case threshold (*n* = 28, r = 0.090, *p* = 0.649) or the ≥10-case threshold (*n* = 19, r = 0.063, *p* = 0.797). However, formal interaction testing did not demonstrate a significant difference in discovery year-onset relationship between the two groups (interaction *p* = 0.125 and *p* = 0.177 for ≥5-case and ≥10-case analyses, respectively).

Separately, we assessed if syndromic-association status itself showed a temporal relationship with year of RP association. Of the 105 RP-associated genes, 34 were classified as having syndromic associations (Table 5). Descriptively, genes with syndromic associations were most represented in the middle discovery eras (from 2001 to 2015); however, no significant temporal association was identified by either point-biserial correlation with year of discovery (r = 0.014, *p* = 0.8895) or by chi-square comparison across discovery eras (χ^2^(6) = 10.31, *p* = 0.1123). The full list of genes and their corresponding clinical features and syndromic associations are listed in Supplementary Table S2.

## 4. Discussion

**Figure 5:**
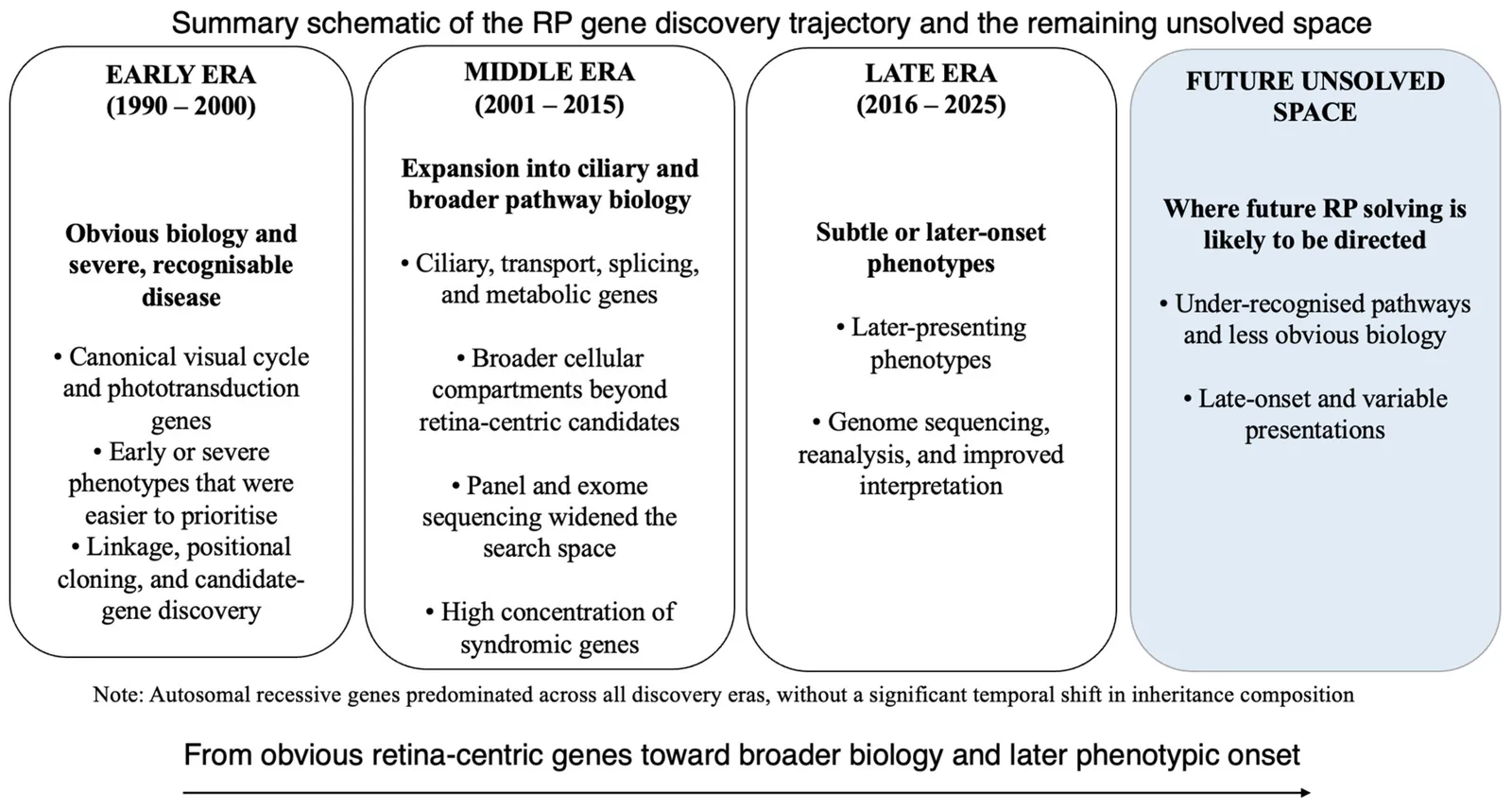
**Summary schematic of RP gene discovery eras and respective gene characteristics.** For visual summary, the RP discovery timeline was grouped into an early era (1990–2000), middle era (2001–2015), and late era (2016–2025), corresponding to the chronological bins in Table 5. Early discovery favoured canonical retina-centric genes and severe recognisable phenotypes, whereas later discovery broadened into ciliary and wider cellular pathways, as well as subtler or later-onset presentations. The remaining unsolved space is therefore proposed to be enriched for under-recognised biology and late-onset or variable disease.

One of the clearest signals in the present study was the distinction between genes identified earlier and those found later. Three findings were particularly informative. First, the biological composition of RP gene discovery changed significantly across eras, with earlier periods primarily enriched for canonical visual cycle and phototransduction genes and later periods demonstrating a broader representation of ciliary, transport, metabolic, and less-defined categories. Second, later discovery year was associated with later mean age of symptom onset at the gene level. Third, while autosomal recessive genes remained prominent across the discovery timeline, the absence of a significant temporal shift in inheritance suggests that future gains in RP solving are unlikely to depend on inheritance pattern alone. This is illustrated in summary Figure 5.

Earlier discovery periods were relatively enriched for visual cycle and phototransduction genes, which were more biologically intuitive, retina-centric, and typically associated with recognisable photoreceptor dysfunction, while later phases increasingly included genes involved in broader cellular pathways. This is reflected in early examples such as *PDE6B*, which was reported as only the second phototransduction gene after rhodopsin to be implicated in classic recessive RP [22], and *RPE65*, which was considered an attractive candidate gene because of its retinal pigment epithelium-specific expression and central role in chromophore recycling and photoreceptor viability [23]. In contrast, later eras broadened into genes involved in ciliary biology, splicing, transport, metabolism, and other less immediately obvious pathways, suggesting a transition from readily prioritised retinal biology toward broader cellular mechanisms that were more difficult to connect to RP using earlier approaches. Discovery of such genes may have required broader sequencing datasets and stronger cross-cohort aggregation before a robust disease association could be established [24]. However, functional categorisation necessarily simplifies genes with pleiotropic or incompletely characterised roles, and assignment of a single dominant category may not fully capture the biological complexity of many RP-associated genes.

This biological broadening likely also reflects the technologies available in each RP discovery wave. Early discoveries were made largely through linkage analysis, positional cloning, and candidate-gene sequencing, approaches that depended heavily on large informative pedigrees, strong segregation, and biologically plausible targets. Later waves of panel sequencing, exome sequencing, and whole-genome sequencing opened a much broader search space [10], while reanalysis pipelines, improved annotation, and newer long-read approaches increased sensitivity to difficult-to-detect variant classes and to structural, copy-number, and non-coding contributors. An example is the *EYS* gene, whose discovery report described it as spanning approximately 2 Mb [25], making it one of the largest human genes and the largest retinal dystrophy gene described at the time. That exceptional size likely contributed to the technical difficulty of comprehensive interrogation in older sequencing paradigms [26]. Likewise, homozygosity mapping and exome sequencing have been shown to accelerate the discovery of cilium-related genes [27]. The later appearance of broader pathway classes in RP therefore likely reflects not only underlying disease biology, but also the evolving technical capacity of genomic medicine. Later-discovered genes may not simply represent a residual tail of discovery, but instead may represent genes that became identifiable only once the necessary technological and interpretive frameworks were available. Therefore, part of the observed temporal shift may reflect not only intrinsic disease biology but also the expanding technical capacity to interrogate larger genes, noncoding regions, and structurally complex loci, rather than a purely biological transition in disease mechanisms.

The use of gene-discovery chronology as a means to understanding the evolution of genetic disease architecture is not unique to RP. In epilepsy, for instance, genetic discovery has been characterised by successive phases progressing from highly informative familial epilepsies predominated by ion-channel biologies, to substantially broader genetic and biological heterogeneity after the introduction of next-generation sequencing (NGS) [28]. Similarly, waves of gene discovery in Charcot-Marie-Tooth disease have linked successive waves of gene discovery to advances in genomic sequencing, with an accompanying expansion of recognised disease mechanisms [29]. Furthermore, at a broader level, systematic reconstruction of gene-disease discovery across more than 4000 rare monogenic diseases demonstrated a correlation between discovery rates and major technological developments, including Sanger sequencing, polymerase chain reaction (PCR) and NGS [30]. As such, although these studies did not examine the same combination of phenotypic and gene-level variables in the present study, they support the broader principle that the historical trajectories of disease-gene discovery may reflect underlying biological architecture and prevailing technologies and ascertainment strategies available at a particular time.

The gene-level association between later discovery year and later mean age of symptom onset is also informative. A reasonable interpretation is that genes associated with severe, early, or otherwise clinically conspicuous disease were more likely to be identified earlier within prevailing discovery paradigms, whereas later presenting or more variably expressed phenotypes remained unsolved for longer. Genes linked to severe, early-onset, or highly penetrant disease are more likely to be recognised earlier because affected individuals present sooner, family segregation is clearer, and the phenotype is more conspicuous [8,12,31,32]. However, given the approximate nature of onset data and potential reporting bias, this relationship should be interpreted as indicative rather than definitive.

This is exemplified by early-discovered genes such as *PDE6G* and *RHO* [33,34], where pathogenic variants were identified in large pedigrees, as well as by genes causing Leber congenital amaurosis, a severe phenotype presenting at birth or infancy that is inherently easier to recognise early in the disease course. In contrast, later-presenting or more variably expressed disease genes may be less amenable to older or more traditional linkage-based or candidate-gene approaches [31,35], particularly in small recessive pedigrees where traditional linkage may perform poorly, or in genes such as *PRPF31*, where incomplete penetrance can obscure otherwise clean segregation patterns [36]. Thus, the temporal association observed here should not be interpreted to mean that later discovery causes milder disease, but rather that the chronology of discovery partly reflects phenotypic recognisability. In practical terms, unresolved RP may be enriched for less clinically obvious, later-presenting, or more heterogeneous phenotypes that were harder to prioritise in earlier discovery paradigms; however, this inference reflects historical discovery patterns and should not be interpreted as predictive at the level of individual unsolved cases.

The inheritance findings should be interpreted more cautiously. Although autosomal recessive genes predominated descriptively throughout the RP discovery timeline, gene-level inferential testing did not demonstrate a significant temporal shift toward autosomal recessive inheritance. This suggests that recessive disease has remained an important and consistent component of RP genetics across discovery eras, rather than representing a distinctly later wave of gene discovery. At the same time, while mixed-inheritance classifications were confined to genes discovered before 2010 in the present dataset, this should not necessarily be interpreted as evidence that mixed modes of inheritance have disappeared among more recently identified RP genes. Rather, this could partly reflect a temporal ascertainment effect inherent to retrospective gene-level classifications. Discovery year was assigned from the earliest RP-associated report, while inheritance status reflects subsequently accumulated evidence for that gene. Since different pathogenic variants within the same RP-associated gene can produce disease through different modes of inheritance [35], earlier-discovered genes have had a substantially longer period in which additional pedigrees and inheritance patterns could be recognised. More recently discovered genes may therefore remain classified according to the inheritance pattern represented in their initial or currently available reports. Hence, given the small number of mixed-inheritance genes overall, this descriptive pattern should be interpreted cautiously. Taken together, these findings suggest that inheritance pattern alone is unlikely to define the remaining unsolved RP discovery space.

Nevertheless, current genetic techniques remain important for the identification of new AR genes. Multiple RP and IRD papers and reviews have suggested that recessive gene identification is strongly enabled by autozygosity or homozygosity mapping in consanguineous pedigrees combined with sequencing. For example, one severe early-onset recessive RP study used genome-wide homozygosity mapping in a consanguineous extended family to localise a shared interval before identifying a homozygous splice-site variant that segregated with disease [33]. Homozygosity mapping has been shown to display significant efficiency and practical yield in detecting novel recessive RP variants [37]. Exome-based Mendelian gene studies have similarly suggested a relative advantage in recessive disorders, as filtering for rare homozygous or compound heterozygous variants can narrow the candidate space more readily than in many dominant or genetically heterogeneous settings [38,39]. These observations suggest that while recessive genes remain central to RP genetics, the unresolved discovery space is likely to be shaped not by inheritance pattern alone, but also by additional factors such as phenotype subtlety, pathway breadth, population-specific variation, and variant detectability.

These observations may suggest that future RP gene discovery may increasingly depend on distinguishing cases remaining unsolved due to previously undetected variation in established genes from those genuinely harbouring novel disease genes. Emerging genomic approaches may facilitate this distinction. For example, long-read sequencing can improve resolution of structurally complex, non-coding, splice-altering and difficult-to-phase variation, while optical genome mapping (OGM) can provide a complementary approach for identifying large and complex structural rearrangements. For instance, OGM has revealed previously overlooked structural variants disrupting established retinal disease genes [11,40]. Such technologies may therefore help increase diagnostic yield without necessarily increasing the number of novel RP genes and refine the residual unsolved cohort where genuine new gene discovery is most likely.

For cases remaining unsolved despite comprehensive DNA-level interrogation, functional and multi-omic approaches become increasingly important. Transcriptomic analysis can help identify aberrant splicing patterns, while proteomic and metabolomic profiling may reveal disturbed molecular pathways and provide functional evidence for prioritising candidate genes [41]. This may be particularly relevant to broader ciliary and metabolic pathways prominently seen in later RP discovery timelines in the present study. Furthermore, as disease-relevant retinal tissue is rarely available clinically, patient-derived induced pluripotent stem cells and retinal organoids may provide a human retinal context in which candidate variants, genes and downstream molecular effects can be assessed [42]. As such, future RP gene discovery may increasingly depend on the integration of complementary genomic and functional approaches as opposed to reliance on greater sequencing depth alone.

A supplementary gene-level analysis identified 34 RP-associated genes with reported syndromic associations but did not show a significant association between syndromic status and year of discovery. Although this does not support a simple model in which genes with syndromic associations were systematically discovered later, it remains consistent with the broader expansion of RP genetics beyond isolated retina-centric disease into multisystem and ciliopathy-associated biology [43]. One possible explanation is that genes with syndromic associations entered the RP discovery landscape through different routes, where some were recognised relatively early because of distinctive clinical syndromes, whereas others emerged later through broader sequencing approaches and phenotypic expansion, when retinal degeneration was appreciated as part of a wider systemic presentation [43,44]. Syndromic status alone may therefore be insufficiently granular to show a clear chronological pattern.

Stratification by syndromic association status further showed that the positive relationship between later discovery year and later mean age of symptom onset remained evident among non-syndromic genes, while no significant temporal association was detected among genes with syndromic associations. The formal interaction testing, however, did not show a significant difference between the two groups; hence, these findings should not be interpreted as evidence of distinct temporal trajectories. A smaller number and greater phenotypic heterogeneity of genes with syndromic associations may limit detection of a gene-level relationship within this subgroup. This interpretation should also be made cautiously, as the present classification was assigned at the gene level and does not distinguish genes associated exclusively with syndromic RP from genes reported in both isolated and syndromic RP, or from genes with variable extraocular involvement [45].

This study has several limitations. As a literature-derived analysis, it reflects publication practices as well as underlying disease biology, and the amount of evidence supporting each RP gene varies substantially across reports. Discovery year is also an imperfect proxy for biological detectability, as it is shaped not only by phenotype and gene characteristics but also by evolving sequencing technologies, analytic methods, and study populations. Functional categories were assigned according to the principal known role of each gene, which may oversimplify genes with pleiotropic or incompletely established functions. In addition, age-of-symptom-onset data extracted from published reports may be subject to recall bias, inconsistent reporting, and approximation when descriptive categories such as infancy or childhood were converted into numerical values. Accordingly, onset analyses should be interpreted as approximate indicators of phenotypic onset rather than precise quantitative measurements. In addition, reliance on curated gene resources such as RetiGene introduces potential bias related to gene inclusion and classification, which may evolve and influence apparent discovery patterns.

A strength of the present approach is that it treats the history of RP gene discovery as informative. Although publication-era effects and reporting heterogeneity cannot be fully eliminated, the observed temporal broadening from canonical visual-cycle and phototransduction genes toward wider cellular pathways, together with the association between later discovery year and later symptom onset, provides a useful framework for thinking about what may remain unsolved. This literature-derived study therefore allows a move beyond mere description to generate plausible forward-looking hypotheses about the remaining unsolved RP discovery space, without directly sequencing unsolved cohorts.

## 5. Conclusions

In this literature-derived analysis, the history of RP gene discovery suggested an apparent progression from early identification of clinically conspicuous, biologically intuitive genes toward later discovery of broader pathway classes and later-presenting phenotypes. Earlier discovery periods were relatively enriched for canonical visual-cycle and phototransduction genes, whereas later periods showed wider representation of ciliary, transport, metabolic, and other less canonical biological compartments. Although autosomal recessive genes predominated descriptively across the discovery timeline, gene-level inferential analysis did not demonstrate a significant temporal shift toward autosomal recessive inheritance. These findings suggest that the remaining unsolved RP discovery space may reflect non-random patterns shaped by both biological and discovery-related factors. Future gains are therefore likely to depend on improved detection of less clinically conspicuous or later-presenting phenotypes and continued exploration of under-appreciated biological pathways.

## Figures and Tables

**Table 1:** Descriptive distribution of inheritance patterns across discovery eras at the gene level.

Discovery Era	AD, *n* (%)	AR, *n* (%)	XL, *n* (%)	AD-AR, *n* (%)	AD-XL, *n* (%)	Total, *n*
1990 to 1999	4 (18.2)	12 (54.5)	1 (4.5)	4 (18.2)	1 (4.5)	22
2000 to 2004	4 (19.0)	14 (66.7)	0 (0.0)	3 (14.3)	0 (0.0)	21
2005 to 2009	2 (12.5)	12 (75.0)	1 (6.3)	1 (6.3)	0 (0.0)	16
2010 to 2014	2 (9.1)	20 (90.9)	0 (0.0)	0 (0.0)	0 (0.0)	22
2015 to 2019	1 (10.0)	9 (90.0)	0 (0.0)	0 (0.0)	0 (0.0)	10
2020 to 2025	1 (7.1)	10 (71.4)	3 (21.4)	0 (0.0)	0 (0.0)	14
Total	14 (13.3)	77 (73.3)	5 (4.8)	8 (7.6)	1 (1.0)	105

**Note:** All 105 RP-associated genes were included, with mixed inheritance categories retained for descriptive analysis. Percentages are row percentages within each discovery era.

**Table 2:** Gene-level distribution of autosomal recessive versus non-autosomal recessive inheritance across discovery eras.

Discovery Era	Non-AR (AD/XL), *n* (%)	AR, *n* (%)	Total, *n*
1990 to 1999	5 (29.4)	12 (70.6)	17
2000 to 2004	4 (22.2)	14 (77.8)	18
2005 to 2009	3 (20.0)	12 (80.0)	15
2010 to 2014	2 (9.1)	20 (90.9)	22
2015 to 2019	1 (10.0)	9 (90.0)	10
2020 to 2025	4 (28.6)	10 (71.4)	14
Total	19 (19.8)	77 (80.2)	96
Pearson chi-square = 3.929, df = 5, *p* = 0.560			

**Note:** Percentages are row percentages within each discovery era. Mixed-inheritance genes were excluded from inferential analysis.

**Table 3:** Year of discovery by functional category with corresponding genes.

Protein or Structure Category	Genes, *n*	Corresponding Genes	Mean Discovery Year	Median Discovery Year	Range
Visual cycle/phototransduction	30	*AIPL1*, *CDHR1*, *CERKL*, *CNGA1*, *CNGB1*, *CRB1*, *CRX*, *GNAT1*, *GUCA1A*, *GUCY2D*, *IMPG1*, *IMPG2*, *LRAT*, *MERTK*, *NR2E3*, *NRL*, *PDE6A*, *PDE6B*, *PDE6G*, *PRCD*, *PRPH2*, *RAX2*, *RBP3*, *RCBTB1*, *RDH12*, *RHO*, *RLBP1*, *RPE65*, *SAG*, *TOPORS*	2001	2000	1990 to 2016
Lipid metabolism	1	*CYP4V2*	2004	2004	2004 to 2004
Splicing factor	7	*DHX38*, *KLHL7*, *PRPF3*, *PRPF31*, *PRPF4*, *PRPF8*, *SNRNP200*	2007	2009	2001 to 2014
Cilium	35	*AHI1*, *ARL2BP*, *ARL3*, *ARL6*, *BBS1*, *BBS2*, *BBS9*, *CC2D2A*, *CEP162*, *CEP250*, *CFAP20*, *CFAP410/C21orf2*, *CFAP418*, *CLRN1*, *DYNC2H1*, *EYS*, *FAM161A*, *IFT140*, *IFT172*, *INPP5E*, *KIAA1549*, *KIZ*, *MAK*, *OFD1*, *PCARE/C2orf71*, *RP1*, *RP1L1*, *RP2*, *RPGR*, *SPATA7*, *TBC1D32*, *TMEM216*, *TTC8*, *TULP1*, *USH2A*	2009	2009	1996 to 2023
Nucleotide metabolism	8	*C19orf44*, *HK1*, *IDH3A*, *IDH3B*, *IDH3G*, *IMPDH1*, *MVK*, *PDSS1*	2011	2011	1992 to 2025
Autophagic and endolysosomal pathway	4	*CLN3*, *HGSNAT*, *SLC66A1*, *UBAP1L*	2012	2014	1995 to 2024
Transmembrane transport	12	*BEST1*, *CLCC1*, *CREB*, *DHDDS*, *FLVCR1*, *POMGNT1*, *REEP6*, *SLC24A1*, *SLC37A3*, *SLC4A7*, *SLC7A14*, *ZNF408*	2013	2014	1998 to 2025
Nucleotide/lipid metabolism	3	*COQ2*, *COQ5*, *COQ8B*	2016	2018	2005 to 2024
Cytoskeleton	2	*AGBL5*, *ARHGEF18*	2016	2016	2015 to 2017
Transcription factor	1	*BCOR*	2022	2022	2022 to 2022
Indeterminate/unknown	2	RP17 locus, Xq27.1 locus	2023	2023	2020 to 2025
Overall comparison: ANOVA *p*-value = 4.26 × 10^−6^ Kruskal–Wallis *p*-value = 5.98 × 10^−5^					

**Note:** Overall comparison across categories was assessed using one-way analysis of variance and Kruskal–Wallis testing.

**Table 4:** Proportions of functional categories affected across discovery eras.

Protein/Structure Category	1990–1994	1995–1999	2000–2004	2005–2009	2010–2014	2015–2019	2020–2025
Autophagic and endolysosomal pathway (AEL)	0 (0.0%)	1 (5.9%)	0 (0.0%)	1 (6.2%)	0 (0.0%)	0 (0.0%)	2 (14.3%)
Cilium	0 (0.0%)	5 (29.4%)	6 (28.6%)	7 (43.8%)	11 (50.0%)	3 (30.0%)	3 (21.4%)
Cytoskeleton	0 (0.0%)	0 (0.0%)	0 (0.0%)	0 (0.0%)	0 (0.0%)	2 (20.0%)	0 (0.0%)
Lipid metabolism	0 (0.0%)	0 (0.0%)	1 (4.8%)	0 (0.0%)	0 (0.0%)	0 (0.0%)	0 (0.0%)
Nucleotide metabolism/Lipid metabolism	0 (0.0%)	0 (0.0%)	0 (0.0%)	1 (6.2%)	0 (0.0%)	1 (10.0%)	1 (7.1%)
Nucleotide metabolism	1 (20.0%)	0 (0.0%)	1 (4.8%)	2 (12.5%)	1 (4.5%)	1 (10.0%)	2 (14.3%)
Splicing factor	0 (0.0%)	0 (0.0%)	3 (14.3%)	2 (12.5%)	2 (9.1%)	0 (0.0%)	0 (0.0%)
Transcription factor/Chromatin remodelling	0 (0.0%)	0 (0.0%)	0 (0.0%)	0 (0.0%)	0 (0.0%)	0 (0.0%)	1 (7.1%)
Transmembrane transport	0 (0.0%)	1 (5.9%)	1 (4.8%)	0 (0.0%)	5 (22.7%)	2 (20.0%)	3 (21.4%)
Indeterminate/unknown	0 (0.0%)	0 (0.0%)	0 (0.0%)	0 (0.0%)	0 (0.0%)	0 (0.0%)	2 (14.3%)
Visual cycle and phototransduction (VCPh)	4 (80.0%)	10 (58.8%)	9 (42.9%)	3 (18.8%)	3 (13.6%)	1 (10.0%)	0 (0.0%)
Total genes (bin denominator)	5	17	21	16	22	10	14

**Note:** Data are shown as *n* (%) of genes per 5-year discovery bin; denominators are provided in the final row. Each gene contributed one observation. Percentages are calculated within bins and may not total 100% due to rounding.

**Table 5:** Descriptive table of genes with syndromic associations across the RP discovery timeline and temporal association analyses.

Discovery Era	Total Genes	Genes with Syndromic Associations	% with Syndromic Associations
1990–1995	9	2	22.2
1996–2000	16	2	12.5
2001–2005	20	9	45.0
2006–2010	23	9	39.1
2011–2015	15	8	53.3
2016–2020	10	2	20.0
2021–2025	12	2	16.7
Overall	105	34	32.4
Point-biserial correlation between syndromic-association status and year of discovery: Pearson’s r = 0.014, *p* = 0.8895 Chi-square comparison across discovery eras, χ^2^(6) = 10.31, *p* = 0.1123			

**Note:** 34 genes were classified as having syndromic associations if their broad phenotype category in the original dataset was coded as “Both”. No statistically significant association was identified between syndromic-association status and year of discovery.

## Data Availability

No new patient-level data were generated. All data presented were derived from published sources as described in this article.

## Supplementary Materials

The following supporting information can be downloaded at: https://www.mdpi.com/article/10.3390/genes17080940/s1, Supplementary Table S1: Gene-level support table summarising the number of cases contributing to age-of-symptom onset and mean, median and standard deviation of age of symptom onset for each gene, as well as PMID links used from the original RetiGene database and supplementary PubMed articles for additional age-of-symptom-onset extraction. Supplementary Table S2: Comprehensive syndromic and extra-ocular phenotype characteristics of RP-associated genes.

## Abbreviations

The following abbreviations are used in this manuscript:

IRD	Inherited Retinal Dystrophies
RP	Retinitis Pigmentosa
AD	Autosomal Dominant
AR	Autosomal Recessive
XL	X-Linked
PMID	PubMed Identifier
ANOVA	Analysis of Variance
PCR	Polymerase Chain Reaction
NGS	Next-Generation Sequencing
OGM	Optical Genome Mapping
