# Supplementary material for: The Trajectory of Gene Discovery in Retinitis Pigmentosa
Source: Genes (Basel). 2026 Aug 12;17(8):940. doi: 10.3390/genes17080940 (PMC13512129; doi:10.3390/genes17080940)
Supplement: Supplementary file 1 [file genes-17-00940-s001.zip › genes-4451861-supplementary.pdf]

Supplementary Table S1. Gene-level support for age-of-symptom-onset analyses and PMID sources referenced

For each RP-associated gene, the table summarises year of discovery, total extracted cases, number of cases with extractable age-of-symptom-onset data, and gene-level onset statistics.

**Total genes**	105
**Genes with ≥5 onset cases**	83
**Genes with ≥10 onset cases**	66

Gene	Year of gene discovery	Cases with extractable onset data	Mean age of symptom onset (years)	Median age of symptom onset (years)	SD of symptom onset (years)	PMIDs of RetiGene articles used	Supplementary PubMed article PMIDs used
NRL	1990	21	7.5	8	4.8	21981118, 15591106	12796249
RHO	1990	13	12.5	15	10.3	21174529, 37712069	26887858, 8240108, 11520753, 16170112
PRPH2	1991	27	34.4	38	16.0	32531846, 8019570	
MVK	1992	7	17.6	15	16.0	24084495, 28095071	25390116, 16435210
PDE6B	1993	22	10.8	8.5	10.1	8394174	7599633, 9543643, 30646425, 33177553
CLN3	1995	17	14.9	16	11.8	36912596	33507216
CNGA1	1995	11	4.1	4	0.3	25268133, 30652268	27391953
PDE6A	1995	17	5.6	5	1.9	21039428	26321862, 30619975, 29118501, 26806561, 35033039
SAG	1995	21	18.8	4	18.4	28549094	31257036, 33047631
GNAT1	1996	2	10	10	8.5	26472407, 27977773	
GUCY2D	1996	12	3.8	0.5	6.0	12623820, 37327959, 16272259	16205573

Gene	Year of gene discovery	Cases with extractable onset data	Mean age of symptom onset (years)	Median age of symptom onset (years)	SD of symptom onset (years)	PMIDs of RetiGene articles used	Supplementary PubMed article PMIDs used
RPGR	1996	49	9.7	6	8.5	36276946, 32012938, 33712029, 12920075, 36017691	
CRX	1997	10	0.9	1	1.2	9931337, 11748859, 9792858	20079931
RLBP1	1997	10	4	4	0	11449319	10102298, 11176989
RPE65	1997	10	1.3	0.5	1.7	9326941, 11264131, 10090910	16272259, 15557452, 11462243
BEST1	1998	10	11.6	8	10.8	19853238, 29503890	35186678, 38841332, 40724865
GUCA1A	1998	1	20	20	NA	32298085	
RP2	1998	12	8.5	4	8.9	36882936	9895243, 10634633, 11020419, 16969763
TULP1	1998	13	2.9	4	2.4	17620573, 15024725 , 17962469, 10711677	29843741, 22605927
USH2A	1998	24	20.3	20	8.5		22004887
CRB1	1999	12	6.8	5	7.8	11389483, 11231775, 21484995	16272259, 11559858
RP1	1999	25	22.5	24	13.4	15863674	23077400, 11527933
AIPL1	2000	12	1.2	0.5	2.8	10615133, 33067476	38880373, 11548141, 11910553, 12881340
MERTK	2000	12	7.3	7	3.7	11062461	20300561
NR2E3	2000	15	12	12	6.5	17564971, 22605927	
BBS2	2001	21	10.8	4	17.3	25541840, 33777945	
CLRN1	2001	12	6.3	4	3.8	21310491	15521980, 12834121, 18281613, 23304067
CNGB1	2001	22	6.7	4	8.3	11379879, 15557452	32613137, 24339724, 28056120
LRAT	2001	17	2.1	2	1.8	31448181, 18055821, 17011878, 11381255, 22570351	
POMGNT1	2001	10	11.5	7	12.8	24282183, 26908613, 27391550, 33175337	38137617, 17906881

Gene	Year of gene discovery	Cases with extractable onset data	Mean age of symptom onset (years)	Median age of symptom onset (years)	SD of symptom onset (years)	PMIDs of RetiGene articles used	Supplementary PubMed article PMIDs used
PRPF31	2001	69	18.4	14	14.7		32014492, 18177735, 12714658
PRPF8	2001	53	12.2	10	8.5	20232351, 22039234	
BBS1	2002	14	11.6	10	9.9	23143442	
IMPDH1	2002	14	16.2	13	13.7	11875050, 16384941, 26720483	15851576
PRPF3	2003	15	9.3	7	5.9	27886254, 28848678	18412284, 15085354, 20309403
TTC8	2003	8	7.4	3.5	6.5	26195043, 20451172	37322672
AHI1	2004	5	17.3	20	15.9	28442542	21458016, 25356976
ARL6	2004	3	5	4	1.7	31736247	36550847, 38927698
CERKL	2004	16	20	20	7.8	18515597, 29068140, 37331655	
CYP4V2	2004	10	24.6	30	11.1	22693542	31872526, 35814500, 40702517
IMPG1	2004	2	30	30	18.4	32817297	40269797
RAX2	2004	7	32.1	35	16.0	30377383, 25789692	
RDH12	2004	11	5.8	4	3.8	15258582, 19140180, 32322264, 15258582	
BBS9	2005	6	19	14	17.0	33771153	38534779, 32686083, 33777945
COQ2	2005	4	24.8	27.5	15.5		35112026, 36266294
HGSNAT	2006	17	34.5	30	18.2	25859010	32347150, 32770643
PRCD	2006	3	6.3	6	2.5	16938425, 20507925	23805042
PDSS1	2007	6	22.5	21.5	16.1	36266294	
TOPORS	2007	10	26.4	27.5	12.0	17924349	18509552, 19183411

Gene	Year of gene discovery	Cases with extractable onset data	Mean age of symptom onset (years)	Median age of symptom onset (years)	SD of symptom onset (years)	PMIDs of RetiGene articles used	Supplementary PubMed article PMIDs used
CC2D2A	2008	1	0	0	NA	30267408	
EYS	2008	15	25.9	21	16.2	18976725	20537394
IDH3B	2008	0	NA	NA	NA	(No extractable age-of-onset cases)	(No extractable age-of-onset cases)
DYNC2H1	2009	6	18.1	19.5	16.5	32753734, 33755199	
INPP5E	2009	13	9	4	8.2	34188062	
KLHL7	2009	5	53	53	0	22084217, 27392078	
OFD1	2009	2	4	4	2.8	29843741	28191358
RBP3	2009	10	9.4	0.5	20.4	19074801, 37806543	25766589
SNRNP200	2009	47	13	10.5	8.5	19710410, 23029027, 31260034	16612614
SPATA7	2009	10	0.9	0	1.6	19268277, 21310915,	23300508
CDHR1	2010	16	18.3	18	11.4	20087419, 28765526 , 35627310	35656873
FAM161A	2010	13	14.5	15	10.3	20705278, 31236346	24520187, 26113502, 26246154
FLVCR1	2010	21	6.2	4	9.3	21070897, 21267618, 29192808, 30656474	
IMPG2	2010	20	8.2	5.8	5.4	20673862	24876279
PCARE/c2orf71	2010	10	10.5	8	8.3	20398884, 20398886	21412943, 21792230, 25544989
PDE6G	2010	3	4	2	3.5	20655036	39160471
RP1L1	2010	10	29.7	31	16.5	23281133, 30025130	40172514, 31236346
SLC24A1	2010	5	16.2	7	16.8		35486108
TMEM216	2010	54	6.0	4	5.8		39191256

Gene	Year of gene discovery	Cases with extractable onset data	Mean age of symptom onset (years)	Median age of symptom onset (years)	SD of symptom onset (years)	PMIDs of RetiGene articles used	Supplementary PubMed article PMIDs used
DHDDS	2011	19	27.2	26	8.6		25255364
MAK	2011	15	34.2	40	14.5	21835304	25385675
CFAP418	2012	11	11.9	15	7.2	27008867, 22177090, 24618324, 25802487, 26854863	23788369
IFT140	2012	16	13.7	10.5	11.9	26968735, 36393898, 22503633	26216056
ARL2BP	2013	11	21.1	23	9.5	36507858, 31425546, 27790702, 23849777, 30210231	38649918
IFT172	2013	6	6.3	5	4.3	24140113, 25168386, 26763875	40370963
ZNF408	2013	7	33.1	30	15.9	25882705, 28095122	34259982
CEP250	2014	10	14.8	10	12.4	30998843, 24780881	30459346, 34223797, 38674450, 39610034
DHX38	2014	16	3.9	4	0.3	30208423, 35719279, 24737827	
HK1	2014	17	15.2	13	7.9	25190649	25316723
KIZ	2014	10	27.6	21.2	12.1		32052671, 31556760
PRPF4	2014	6	17.7	20	9.6	24419317, 25383878	37264419
SLC7A14	2014	15	19.4	18	17.4	24670872	27028480, 30924391
AGBL5	2015	10	10.3	8.2	8.1	26720455, 27842159	27764769, 30925032
CFAP410/c21orf2	2015	10	10.8	4	15.2	36950666, 26294103	27548899, 31431468, 33307614
ARL3	2016	10	21.7	17.5	15.3	26964041, 30932721, 33748123	41828587, 40037334
RCBTB1	2016	4	24	17.5	15.4	27486781	27608171
REEP6	2016	10	18.6	17	11.9	27889058, 31725169, 29120066, 33917198	
ARHGEF18	2017	3	26.3	29	5.5	28132693	

Gene	Year of gene discovery	Cases with extractable onset data	Mean age of symptom onset (years)	Median age of symptom onset (years)	SD of symptom onset (years)	PMIDs of RetiGene articles used	Supplementary PubMed article PMIDs used
IDH3A	2017	10	6.4	6.5	3.8	28412069, 31012789	30058936, 40993038
CLCC1	2018	5	10.2	10	1.3	30157172	
COQ5	2018	2	31.5	31.5	24.7	36266294	
KIAA1549	2018	3	27	28	18.5	30120214	
RP17 locus	2020	17	28.1	25	13.2	33022222	
SLC4A7	2020	3	20	16	18.3	35486108, 32594822	
BCOR	2022	5	8	8	0	36070393	
CFAP20	2022	1	18	18	NA	36329026	
SLC37A3	2022	5	45.2	44	11.9	35486108	40069519
SLC66A1	2022	1	30	30	NA	35486108	
CEP162	2023	2	48	48	11.3	36862503	
TBC1D32	2023	4	12	12	9.2	37768732	39930170
COQ8B	2024	5	22.8	26	14.0	39226897, 40450584	
UBAP1L	2024	12	33.9	35.5	19.4	38293907, 38420906	
C19orf44	2025	7	39.1	43	20.4	40079362	
CREB	2025	0	NA	NA	NA	(No extractable age-of-onset cases)	(No extractable age-of-onset cases)
IDH3G	2025	0	NA	NA	NA	(No extractable age-of-onset cases)	(No extractable age-of-onset cases)
Xq27.1 locus	2025	3	4	4	0	39892393	

Supplementary Table S2: Comprehensive syndromic and extra-ocular phenotype characteristics of RP-associated genes.

Gene	Year of first RP association	Syndromic/systemic association	Major extra-ocular organ system(s)	Major extra-ocular clinical features
**MVK**	1992	Mevalonic aciduria / mevalonate kinase deficiency	Immunologic/inflammatory; neurologic	Immunologic/inflammatory: Recurrent childhood febrile crises,<br><br>Neurological: Progressive cerebellar ataxia, psychomotor developmental delay<br><br>Structural anomalies: Distinctive facial dysmorphism, hepatosplenomegaly
**CLN3**	1995	Neuronal ceroid lipofuscinosis / Batten disease	Neurologic, cardiac	Neurological: Progressive cognitive decline, seizures (epilepsy), extrapyramidal and cerebellar motor dysfunction, psychiatric disturbances<br><br>Cardiac: Arrhythmias or cardiomyopathy
**RPGR**	1996	X-linked RP with sinorespiratory disease with or without deafness	Respiratory; auditory	Respiratory: Recurrent chronic rhinosinusitis, sinorespiratory tract infections, bronchiectasis<br><br>Auditory: Sensorineural hearing loss, recurrent otitis media

**USH2A**	1998	Usher syndrome	Auditory	Auditory: Congenital, moderate-to-severe sensorineural hearing loss<br><br>(Vestibular function usually preserved)
**BBS2**	2001	Bardet–Biedl syndrome	Renal; metabolic/endocrine; musculoskeletal; reproductive; neurodevelopmental	Musculoskeletal: Postaxial polydactyly<br><br>Metabolic/endocrine: Early-onset central obesity, endocrine/metabolic dysfunction (type 2 diabetes mellitus, metabolic syndrome)<br><br>Reproductive: Hypogonadism<br><br>Renal: Progressive renal dysfunction<br><br>Neurodevelopmental: Variable cognitive impairment
**CLRN1**	2001	Usher syndrome type IIIA	Auditory	Auditory: Progressive sensorineural hearing loss, variable vestibular dysfunction manifesting later in life
**POMGNT1**	2001	Muscle-eye-brain disease	Neurologic; musculoskeletal	Neurological: Severe neonatal hypotonia ("floppy baby syndrome"), brain malformations (cobblestone lissencephaly)

**BBS1**	2002	Bardet–Biedl syndrome	Renal; metabolic/endocrine; musculoskeletal; reproductive; neurodevelopmental	Metabolic/endocrine: Early childhood truncal obesity<br><br>Skeletal: Postaxial polydactyly (extra fingers or toes),<br><br>Neurological: Cognitive impairment<br><br>Renal: Kidney abnormalities<br><br>Reproductive: Hypogonadism
**TTC8**	2003	Bardet–Biedl syndrome	Renal; metabolic/endocrine; musculoskeletal; reproductive; neurodevelopmental	Metabolic/endocrine: Central obesity, hypogonadism<br><br>Skeletal: Postaxial polydactyly<br><br>Renal: Structural abnormalities<br><br>Neurological: Cognitive impairment, anosmia, neurological motor deficits, neurodevelopmental delays

**AHI1**	2004	Joubert syndrome	Neurologic; renal; hepatic	Neurological: Cerebellar vermis hypoplasia ('molar tooth sign' on MRI)<br><br>Renal: nephronophthisis<br><br>Hepatic: Congenital hepatic fibrosis,<br><br>Skeletal: Facial skeletal dysmorphism<br><br>Respiratory: Neonatal breathing abnormalities (hypopnea/hyperpnea/apnea)
**ARL6**	2004	Bardet–Biedl syndrome type 3	Renal; metabolic/endocrine; musculoskeletal; reproductive; neurodevelopmental	Metabolic/endocrine: Childhood-onset obesity, hypogonadism (micropenis, cryptorchidism, delayed puberty, genital hypoplasia)<br><br>Skeletal: Polydactyly (extra fingers or toes)<br><br>Renal: Kidney structural abnormalities<br><br>Neurological: Cognitive impairment or developmental delay

**BBS9**	2005	Bardet–Biedl syndrome	Renal; metabolic/endocrine; musculoskeletal; reproductive; neurodevelopmental	Metabolic/endocrine: Childhood-onset obesity<br><br>Skeletal: Post-axial polydactyly (extra fingers or toes)<br><br>Neurological: Learning difficulties<br><br>Renal: Kidney abnormalities<br><br>Reproductive: Genital hypoplasia
**COQ2**	2005	Coenzyme Q10 deficiency spectrum	Renal; neurologic; auditory; hematologic/metabolic	Renal: Early-onset or progressive renal dysfunction<br><br>Neurological: Neurological impairment<br><br>Auditory: Sensorineural deafness<br><br>Hematologic/metabolic: Thrombocytopenia, lactic acidosis
**HGSNAT**	2006	Mucopolysaccharidosis type IIIC / Sanfilippo C syndrome	Neurologic; auditory; craniofacial	Neurological: Progressive dementia, behavioural difficulties, sleep disorders, epilepsy<br><br>Auditory: Hearing impairment<br><br>Craniofacial: Coarse facial features

**PDSS1**	2007	Coenzyme Q10 biosynthesis disorder	Auditory; neurologic/developmental; neuromuscular; renal	Auditory: Sensorineural deafness<br><br>Neurologic/developmental: Optic atrophy, developmental delay<br><br>Neuromuscular: Muscular weakness<br><br>Renal: Steroid-resistant nephrotic syndrome
**CC2D2A**	2008	Joubert syndrome	Neurologic; renal; hepatic; musculoskeletal; craniofacial/oral	Neurological: Brain malformations ("molar tooth sign"), hypotonia, ataxia, global developmental delay<br><br>Renal: Cystic kidneys<br><br>Hepatic: Liver fibrosis<br><br>Musculoskeletal: Polydactyly<br><br>Craniofacial/oral: Orofacial abnormalities
**DYNC2H1**	2009	Jeune syndrome / asphyxiating thoracic dystrophy (short-rib polydactyly syndrome)	Skeletal; renal; hepatic; cardiac; respiratory	Skeletal: Thoracic hypoplasia, micromelia, kyphoscoliosis, polydactyly<br><br>Renal: Polycystic kidneys<br><br>Hepatic: Hepatic fibrosis<br><br>Cardiac: Cardiac malformations<br><br>Respiratory: Pulmonary hypoplasia

**INPP5E**	2009	Joubert syndrome	Neurologic; renal; respiratory; metabolic/endocrine; musculoskeletal	Neurological: Cerebellar ataxia, developmental delay, hypotonia<br><br>Respiratory: Abnormal breathing patterns (hyperpnea)<br><br>Metabolic/endocrine: Truncal obesity<br><br>Musculoskeletal: Occasional polydactyly<br><br>Renal: Occasional renal anomalies
**KLHL7**	2009	PERCHING / Bohring–Opitz / Crisponi-spectrum phenotype	Neurodevelopmental; craniofacial; musculoskeletal; respiratory	Neurodevelopmental: Intellectual disability<br><br>Craniofacial: Facial dysmorphism<br><br>Musculoskeletal: Joint contractures<br><br>Respiratory: Respiratory distress

**OFD1**	2009	Joubert / oral-facial-digital spectrum	Neurologic; renal; craniofacial/oral; musculoskeletal; integumentary	Craniofacial/oral: Orofacial abnormalities (lobulated tongue, cleft palate, hypodontia, hypertelorism)<br><br>Musculoskeletal: Brachydactyly, syndactyly, clinodactyly<br><br>Integumentary: Hypotrichosis<br><br>Renal: Polycystic kidney<br><br>Neurological: Brain malformations (Dandy-Walker malformation, intracerebral cysts, corpus callosum agenesis), developmental delay
**FLVCR1**	2010	Posterior column ataxia with retinitis pigmentosa	Neurologic; peripheral nervous/autonomic; musculoskeletal; gastrointestinal	Neurological: Sensory ataxia, proprioceptive loss<br><br>Peripheral nervous/autonomic: Hypomyelinating sensory-autonomic neuropathy<br><br>Musculoskeletal: Scoliosis<br><br>Gastrointestinal: Gastrointestinal dysmotility
**TMEM216**	2010	Joubert syndrome	Neurologic; renal	Neurological: Cerebellar vermis hypoplasia ('molar tooth sign' on MRI), hypotonia<br><br>Renal: Nephronophthisis

**CFAP418**	2012	Bardet–Biedl syndrome	Renal; metabolic/endocrine; musculoskeletal; reproductive; neurodevelopmental	Neurodevelopmental: Mild cognitive or developmental delays<br><br>Metabolic/endocrine: Endocrine dysfunction<br><br>Ciliary/structural: Subtle structural ciliary anomalies
**IFT140**	2012	Short-rib thoracic dysplasia / Mainzer–Saldino syndrome / nephronophthisis	Skeletal; renal; reproductive	Skeletal: Thoracic dysplasia<br><br>Renal: Nephronophthisis<br><br>Reproductive: Male infertility
**ARL2BP**	2013	Ciliopathy with laterality abnormalities	Reproductive; renal; olfactory; laterality-associated systems	Laterality-associated: Situs inversus totalis<br><br>Reproductive: Male infertility<br><br>Renal: Renal anomalies<br><br>Olfactory: Anosmia
**IFT172**	2013	Bardet–Biedl / Mainzer–Saldino / Jeune spectrum	Skeletal; renal; metabolic/endocrine; hepatic	Metabolic/endocrine: Truncal obesity<br><br>Skeletal: Postaxial polydactyly, thoracic dysplasia<br><br>Renal: Renal polycystic disease<br><br>Hepatic: Hepatic involvement

**CEP250**	2014	Usher syndrome	Auditory; metabolic/endocrine; neurodevelopmental; respiratory; pancreatic	Auditory: Post-lingual, progressive sensorineural hearing loss (SNHL)<br><br>Metabolic/endocrine: Diabetes mellitus<br><br>Neurodevelopmental: Intellectual disability<br><br>Respiratory: Pulmonary/lung abnormalities<br><br>Pancreatic: Pancreatic insufficiency
**HK1**	2014	Neurodevelopmental/metabolic disorder	Neurologic; neurodevelopmental; metabolic	Neurodevelopmental: Intellectual disability, structural brain anomalies<br><br>Metabolic: Metabolic dysfunction<br><br>Neurological: Seizures
**AGBL5**	2015	Syndromic retinal disease with hearing loss and developmental delay	Auditory; neurodevelopmental	Auditory: Post-verbal sensorineural hearing loss<br><br>Neurodevelopmental: Developmental delay
**CFAP410**	2015	Axial spondylometaphyseal dysplasia with retinal dystrophy	Skeletal; respiratory/thoracic	Skeletal: Axial spondylometaphyseal dysplasia (SMDAX), short stature, scoliosis, hip dysplasia

**ARL3**	2016	Joubert syndrome	Neurologic; renal; hepatic; skeletal	Neurological: Hypotonia, developmental delay, ataxia<br><br>Renal: Nephronophthisis<br><br>Hepatic: Liver fibrosis<br><br>Skeletal: Skeletal anomalies
**RCBTB1**	2016	Syndromic retinal dystrophy with endocrine and neurodevelopmental manifestations	Endocrine; reproductive; neurodevelopmental	Endocrine: Goiter<br><br>Reproductive: Primary ovarian insufficiency<br><br>Neurodevelopmental: Mild intellectual disability
**TBC1D32**	2023	Oro-facial-digital syndrome type IX	Craniofacial/oral; endocrine; auditory; neurodevelopmental	Endocrine: Hypopituitarism<br><br>Auditory: Sensorineural hearing loss<br><br>Neurodevelopmental: Developmental delay
**COQ8B**	2024	Steroid-resistant nephrotic syndrome	Neurologic; renal	Neurological: Seizures or encephalopathy linked secondary to severe hypertension<br><br>Renal: End-stage renal failure (heavy proteinuria, progressive chronic kidney disease)

Note: Full list of the 34 RP-associated genes classified as having syndromic associations in the original dataset, with corresponding systemic or syndromic clinical features. Genes were classified as having syndromic associations where the original phenotype category was coded as “Both”. Syndromic associations and extra-ocular clinical features were derived from the published literature and curated resources used for the present study, as described in the Methods.
